# Self-reported patient experiences in a peer-support community: What do cancer patients value?

**DOI:** 10.3389/fpsyg.2026.1724101

**Published:** 2026-03-05

**Authors:** Florence Horicks, Teodora Lalova-Spinks, Silke Léonard, Delphine Remy, Brent Taels, Hilde Stevens

**Affiliations:** 1 Institute for Interdisciplinary Innovation in Healthcare (I3h), Faculty of Medicine, Université Libre de Bruxelles, Brussels, Belgium; 2 Pôle Santé, Université Libre de Bruxelles, Brussels, Belgium; 3 Department of Public Health and Primary Care, Faculty of Medicine and Health Sciences, Ghent University, Ghent, Belgium; 4 Centre for IT and IP Law, Faculty of Law, KU Leuven, Leuven, Belgium; 5 Department of Health and Life Sciences, AP University of Applied Sciences and Arts, Antwerp, Belgium; 6 DR Square B.V., Overijse, Belgium; 7 Department of Marketing, Innovation and Organisation, Faculty of Economics and Business Administration, Ghent University, Ghent, Belgium; 8 LUCAS - Centre for Care Research and Consultancy, KU Leuven, Leuven, Belgium

**Keywords:** oncology, online health community, patient value, peer support, self-reported patient experience, transdisciplinarity

## Abstract

**Background:**

Using unguided podcast narratives offers a unique and original opportunity to access patient experiences and understand what patient’s value in their care journey in developing true patient-centered oncology models. Online health communities contain a wealth of information, including unsolicited patient experiences that may go beyond what is captured by guided surveys or patient-reported outcome instruments. This study describes patient experiences reported in a peer support community to gain insight into what patients share amongst each other and what they value.

**Methods:**

A purposive sample of 31 unguided, French-speaking self-reported patient narratives were collected from the podcast “Naitre princesse, devenir guerrière” registered between 2021 and 2024. Episodes included patient (*n* = 30) and tandem patient-professional (*n* = 1) voices, reflecting diverse cancer types, treatment stages, and lived experiences. A transdisciplinary team, including the podcast founder, conducted the thematic analysis to identify patterns of patient values across narratives.

**Results:**

From patient narratives, six core themes of patient value emerged: (peer) support, empowerment, relationship with healthcare professionals, quality of life, cancer as an opportunity, and individuality. Narratives underscored patient value as rooted in reclaiming agency, redefining life priorities, and engaging in meaningful action. Participants revealed the irreplaceable role of peers in providing mutual understanding and solidarity, alongside ambivalent but pivotal relationships with healthcare professionals.

**Conclusion:**

Self-reported narratives from peer communities offer empirical evidence into patients’ priorities and lived realities, revealing what patients identify as meaningful in their cancer pathways. These findings accentuate the importance of transdisciplinary analysis of patient experiences beyond clinical outcomes and physician reports. Online health communities represent a promising but underexplored arena for understanding and integrating patient values into care, research, and policy. Future research should further examine their role in shaping participatory and value-driven health systems.

## Introduction

A cancer diagnosis is a life-altering event that profoundly impacts patients physically, emotionally, psychologically, and socially (Taylor, 2024). Beyond the medical challenges associated with treatment, patients frequently struggle with uncertainty, fear, and a need for support that extends beyond the boundaries of clinical care (Niedzwiedz et al., 2019; Bergerot et al., 2024).

The needs of individuals living with cancer are highly personal, and what patients value during their pathway encompasses their unique beliefs, priorities, and expectations (Kurasz et al., 2022). These values are influenced by a range of cultural, social, and personal factors, which in turn affect treatment preferences and care goals (Armstrong and Mullins, 2017). Concepts such as shared decision-making (Barry and Edgman-Levitan, 2012), goal-oriented care (Jennings and Mold, 2024), and even evidence-based medicine (Lockwood, 2004) increasingly reinforce the importance of incorporating “patient values” into healthcare delivery. Despite this growing recognition, there remains no clear consensus in the academic literature on what constitutes “patient value.” Definitions vary, and practical implementation in care settings is inconsistent. As a result, patient value remains a complex and multifaceted concept, interpreted differently by various stakeholders across the healthcare system (Landon et al., 2021).

At the individual level, patient value is deeply rooted in personal experiences, needs, and relationships. It encompasses the subjective dimensions of living with cancer, how individuals interpret their diagnosis, navigate the emotional and existential challenges, experience shifts in identities, and perceive the alignment (or misalignment) of care with their values, preferences, and life goals (Karimi-Dehkordi et al., 2019). Patient value should reflect not only clinical outcome, but also patients’ priorities, life philosophies and personal backgrounds (Lee et al., 2013). Whereas patient value reflects what matters most to individuals and how well care aligns with their goals and priorities, *patient experiences* capture the concrete interactions and processes through which care is delivered. Self-reported patient experiences provide critical insights into what patients find most meaningful along their cancer pathway, whether it be emotional reassurance, information exchange, or practical guidance. These narratives can inform policy, enhance patient-centered care, and optimize support models to better address unmet needs (Grob et al., 2019). However, capturing and analyzing such experiences requires methodologies that honor the deeply personal and subjective nature of each individual’s story (Sunkureddi et al., 2018). Instruments such as patient-reported outcome measures (PROMs) and patient-reported experience measures (PREMs) have been widely developed and validated, particularly in oncology research (Churruca et al., 2021). These tools have demonstrated benefits, including improved symptom management, enhanced quality of life (QoL), and even reduced mortality (Bull et al., 2019). However, many of these instruments are designed primarily from the clinician’s perspective, often reflecting professional priorities rather than those of patients themselves (Deshpande et al., 2011; Colomer-Lahiguera et al., 2023). Where PROMs quantify patient health outcomes, and PREMs describe care processes, neither of them fully captures patient value, which reflects the personal significance patients attribute to their experiences and outcomes. As a result, the authentic patient voice may be underrepresented in both research and practice. Bridging this gap requires a shift toward co-developing tools and frameworks that genuinely reflect patient values.

Online health communities (OHCs) have become increasingly popular platforms where patients connect with peers, voluntarily share their experiences, and offer mutual support (Zhang et al., 2023). OHCs provide a unique form of emotional and practical help that complements the clinical expertise of healthcare professionals (HCP’s) (Hartzler and Pratt, 2011). Through shared lived experiences, OHCs foster a sense of belonging, validation, and empowerment among participants, helping them navigate the complexities of illness with greater resilience (Jablotschkin et al., 2022). Self-reported patient experiences within OHCs represent raw, unfiltered narratives that are rarely leveraged in academic research. Yet, this user-generated content offers a rich, underexplored resource for understanding patient perspectives in depth. Unlike structured clinical data gathered by PROMs and PREMs, these narratives reflect what patients choose to express, what they find meaningful, challenging, or worth sharing with others in similar situations. Despite their potential, self-reported experiences shared within OHCs remain largely absent from mainstream research methodologies. This gap limits our understanding of what truly matters to patients and how support systems can better align with their lived realities.

This study explores the self-reported experiences and the key elements that patients choose to highlight when they participate in an OHC for cancer patients. Its objective is to uncover what patients value most when coping with cancer, based on what they choose to share with peers. We hypothesize that patients disclose what they personally value and what they believe may benefit others. By analyzing these firsthand accounts, we aim to identify recurring themes that reflect shared values and needs. Ultimately, this research underscores the importance of integrating patients’ voices into the broader landscape of cancer research and care. Doing so can enhance the patient experience, inform more value-driven care models, and shape support mechanisms that are truly responsive to the lived experiences of those affected by cancer. This study aims to identify how patients themselves define what is valuable in their cancer journey, as expressed spontaneously in peer narratives.

## Materials and methods

### Presentation of the online health community

We adopted a transdisciplinary approach by collaborating with a patient (DR), who is both the founder of a peer-support community and the creator of the podcast series *Naître princesse, devenir guerrière*. This online health community (OHC) emerged organically from DR’s initial sharing of her lived experience through a personal blog, which prompted engagement and dialogue from individuals facing similar health challenges. Over time, these interactions evolved into a structured peer-support network, reflecting shared needs for information, emotional support, and collective meaning-making. In response to the growing community and its expressed interests, DR subsequently launched a podcast to facilitate knowledge exchange further, amplify patient voices, and strengthen peer connections within this community.

The podcast, designed and hosted by the patient-founder, aims to inspire, support and inform individuals affected by cancer. It has been actively promoted via social media and published on multiple platforms (Spotify©, Youtube©, Google Podcasts©, and Apple Podcasts©) since 2021, with 122 episodes available at the time of data selection. The episode selection process was co-designed with the patient-founder to ensure alignment with the study’s objectives.

### Data collection

Grounded in transdisciplinary research principles (Pohl et al., 2017), our approach highlights the co-creation of study design and shared execution of research activities by academic disciplines and non-academic stakeholders, including patients. Such collaboration fosters meaningful patient involvement and enhances the relevance and impact of health research.

From the outset, the guiding principle was to explore patient value, what truly matters to individuals in their healthcare journeys. The patient-founder conducted an initial pre-selection of 60 episodes, drawing on her intimate knowledge of the content and including both patients’ and HCPs voices. Subsequently, three researchers from diverse disciplinary backgrounds (nursing, law, and biomedical sciences; SL, TL-S, FH) independently reviewed the episodes using their own criteria, such as the presence of self-reported experiences, relevance to healthcare, diversity of perspectives, and depth of insight.

Following a collaborative discussion, a consensus was reached on a shortlist of 36 episodes, 31 of which featured patient voices from 30 different patients (Supplementary Table S1). In this paper, only patients’ episodes were included. One additional episode conducted by a tandem of a patient and a psychologist was included. The final selection aimed to reflect a broad spectrum of lived experiences, including metastatic cancer, relapses, gender diversity, parenthood, and both uplifting and challenging narratives (see Table 1). Episodes were excluded if they exhibited self-promotion, excessive optimism, misleading claims, narrow entrepreneurial focus, or an overly singular perspective on cancer. This selection process was iterative, reflexive, and grounded in shared values. It centered authentic, diverse patient voices and ensured that the resulting dataset meaningfully contributes to broader reflections on patient experience and value in cancer care.

**Table 1:** Podcast narrators’ characteristics (*N* = 30).

Socio-demographic information	
Gender	
Female	87.1% (*N* = 26)
Male	12.9% (*N* = 4)
Country of residence	
Belgium	13.3% (*N* = 4)
France	86.7% (*N* = 26)
Civil status	
Couple	73.3% (*N* = 22)
Single	10% (*N* = 3)
Unspecified	16.7% (*N* = 5)
Parental status	
Has at least one child	80% (*N* = 24)
No children	16.7% (*N* = 5)
Unspecified	3.3% (*N* = 1)

Clinical information	
Age when first diagnosed	
Mean (years)	38
Range (years)	[11–60]
Unspecified	3
Time between diagnosis and podcast recording	
Mean (years)	6, 26
Unspecified	3
Cancer type	
Breast	50% (*N* = 15)
Heamatological	6.7% (*N* = 2)
Head and neck	6.7% (*N* = 2)
Colorectal	16.7% (*N* = 5)
Pancreatic	3.3% (*N* = 1)
Carcinoma	3.3% (*N* = 1)
Sarcoma	3.3% (*N* = 1)
Kidney	6.7% (*N* = 2)
Lung	3.3% (*N* = 1)
Recurrence of cancer	
Yes	16.7% (*N* = 5)
No	76.7% (*N* = 23)
Metastatic	6.7% (*N* = 2)

### Thematic analysis

The selected episodes were transcribed ad verbatim by a third party. Half of the episodes were randomly assigned for English translation, allowing a non-French-speaking researcher to participate in the coding process. Data analysis followed Braun and Clarke’s six-phase framework for thematic analysis, using a combination of inductive and deductive approaches (Braun and Clarke, 2006).

Initially, open coding was independently applied to three transcripts by three researchers (SL, FH, and TL-S) and the podcast’s patient-founder (DR). This pilot phase was informed by both theoretical frameworks (e.g., patient value, lived experience, concepts of care) and emerging patterns grounded in the data. The team then met to compare codes, resolve discrepancies through discussion, and collaboratively refine the codebook (Supplementary Table S2).

All transcripts were randomly assigned for coding to two researchers (FH, TL-S) and imported into NVivo© software for systematic analysis. The researchers began with an initial reading to familiarize themselves with the content and context. Axial coding was then conducted to organize codes into categories and sub-themes, identifying relationships and patterns across the dataset. This iterative process continued beyond data saturation, due to the upfront selection of a large number of episodes.

Reflexivity was maintained throughout the analysis via ongoing discussions between the two coding researchers, including reflections on the evolving codebook, methodological decisions, and emotional responses throughout the analysis. Including the patient-founder in the analysis complements critical reflexivity by enhancing credibility through iterative dialogue and triangulation. Her experiential knowledge contributed to mitigating blind spots and supporting authentic representation of patient experience.

Analysis workshops were held with the whole research team, including the podcast’s patient-founder, to ensure rigor and inclusivity. These sessions involved reviewing final themes in relation to the original transcripts to ensure alignment and representativeness. The team discussed emerging codes, themes, and interpretations. As a transdisciplinary team, we drew attention to collective reflection, challenged individual assumptions, and integrated diverse disciplinary perspectives to achieve a more nuanced and holistic understanding of the data.

## Results

Narrators’ socio-demographic and clinical characteristics are presented in Table 1. The narrators included patients (*n* = 30) and one tandem patient-professional. Amongst patients’ narratives, there were women (*n* = 26) and men (*n* = 4), ranging in age from 26 to 60 years at podcast recording and living with various types of cancer. Patients were at different stages of their treatment journey, offering diverse perspectives. From these self-reported patient narratives, six core themes were identified as key expressions of patient value, namely peer support, empowerment, patient-HCP relationship, quality of life, finding opportunity and individuality. Table 2 present the six core themes, each of them representing various sub-dimensions, that are further outlined below.

**Table 2:** Core themes of patient value identified within narrators’ self-reported experiences.

Core themes
Peer support
Alleviation of isolation
Validation of emotional experiences
Feeling genuinely understood
Helping others
Empowerment
Empowerment in care pathway
Managing oneself
Patient—HCP relationship
Communication (style and content)
Dynamic of relationship
Trust
Shared-decision making
Therapeutic alliance
Quality of life
Physical symptoms
Psychological toll
Integrative practices and self-care strategies
Survivorship
Finding opportunity in cancer
Personal transformation
Priorities in life
Life choices
Individuality
Personality
Timing
Personal environment

### Patient values

#### Peer support

Peer support emerged as a significant and multifaceted aspect within patients’ self-reported experiences. Many described a profound sense of loneliness associated with cancer, even when surrounded by family, friends, or HCPs. As one participant (P15) articulated, *“Despite the fact that you can be accompanied at the family, friend or professional level, you are alone in the disease, in fact.”* This calls attention to the unique emotional burden of cancer that peer relationships can help mitigate. Through shared experiences, participants found not only comfort and recognition but also a sense of purpose in offering support to others navigating similar challenges. Four subdimensions of peer support were subsequently identified: alleviation of isolation, validation of emotional experiences, feeling genuinely understood, and helping others.

The first subdimension relates to alleviation of isolation. It captures how peer interactions mitigated the profound sense of ‘feeling alone’ often experienced by participants, an isolation not always alleviated by support from family or friends. As P10 described, “*People [from peer community] I did not know sent me [gifts]. It was just incredible […] and it still helps me today.”* While the emotional support of close relatives plays a crucial role in coping with illness, participants underscored that the shared experiences of peers offered a powerful source of strength and hope. This was often expressed through the sentiment, “*If they can do it, I can do it.*” For instance, P10 found encouragement by seeing photos of babies born to other pregnant patients undergoing similar experience, while P26 illustrated how hope can be transmitted within peer groups: *“There are a lot of women who came into this group who had really lost hope, who were quite negative. And other women gave them hope, and it’s crazy how contagious it is.”*

A second subdimension of peer support was the validation of emotional experiences, often described as the reassurance of not being “crazy.” Participants expressed a sense of abnormality or confusion in navigating their illness, questioning whether their emotional responses were “normal” or shared by others. Engaging with peers who had undergone similar experiences provided a sense of legitimacy and normalization. This validation helped participants reframe their emotional struggles as understandable and shared, reducing feelings of alienation and enhancing psychological resilience. As P16 stated: *“It’s really something I would have liked to have been told: you are not alone, there are other people who are going through the same thing as you. And that’s normal, it’s normal and legitimate above all.”*

A third subdimension of peer support was the feeling of being truly understood, a connection rooted in shared experience. Participants noted that only those who had lived *“the same experience, the same things, the same pains” (P17)* could fully grasp the emotional and physical toll of illness. Unlike family or friends, who sometimes responded with discomfort, avoidance or well-intentioned but clumsy remarks, peers were perceived as better equipped to offer empathetic and meaningful support. As P16 recounted: *“[…] People did not know how to deal with me. And it hurts me to hear them say things I did not want to hear at all. […] I shut myself out because I found no comfort in the responses of those close to me.”* This disconnect sometimes led participants to withdraw emotionally from their immediate social circles. Some participants also described feelings of shame or guilt about their illness, as though they were burdening their loved ones. In this context, peer support offered a safe space for emotional expression without fear of causing distress. P17 reflected on this dynamic: “*It’s very difficult to open up to loved ones. I’m afraid of hurting them, […] Of sharing so much pain that they absorb it all. I did not want them to be a sponge for that. […] There’s no one better than a peer in similar circumstances to help another peer.”* These peer relationships allowed for more honest conversations and helped reduce the emotional burden participants felt they imposed on their families.

Finally, a fourth subdimension was the opportunity to help others in ways they themselves had needed. This altruistic drive was often framed as a way to create meaning from their own experience. For some, it even led to a professional reorientation, becoming coaches, writers or expert patients (as further explored below). P18 shared, “*I was alone […]. So I created something, an object, a tool of hope. I created what I would have so desperately needed to hear [at that time].”* Similarly, P24 explained, *“Throughout my entire care journey, I kept thinking, ‘This cannot be’. I do not want any woman to go through what I went through, to face the choices I had to make*.*”*

Despite peer support being a core theme of patient value, not all peer interactions were perceived positively. Some participants found online peer accounts distressing, particularly when they focused on worst-case prognoses, persistent complaints, or emotionally charged exchanges. These contrasting experiences underscore the complex, ambivalent, and reciprocal nature of peer support. This dynamic can be both empowering and emotionally taxing, depending on the context and content of the interactions.

#### Empowerment

Across patients’ self-reported experiences in the podcasts, empowerment emerged as a core theme, represented by two distinct but interconnected subdimensions: on the one hand, empowerment within the care pathway, encompassing patient needs, preferences, decision-making and voice; and on the other hand managing oneself, referring to the reclaiming of control over one’s life through selfcare, mindset, and resilience.

The first subdimension, empowerment in the care pathway, is closely tied to interactions with HCPs. In brief, participants described the importance of being actively involved in decisions about their treatment, resisting a passive role in the care process. They expressed the value of shared decision-making, personalized guidance, and building a relationship of trust with HCPs. This sense of partnership was seen as essential to shaping a care pathway that reflects the patient’s values and preferences. As P31 noted, *“[…] I’m really talking about suffering, because we still find ourselves in a spiral where I realize that I no longer make any decisions, that I am considered a bit incapable of making these decisions. The patient is not solicited at any time.”*

The second subdimension, (self-) empowerment in managing oneself and one’s illness, reflects a strong desire among participants to regain agency and autonomy in the face of their diagnosis. Many expressed frustration with having passively endured treatments and medical decisions, and described a turning point where they chose to reclaim control. P27 shared, *“I told myself, I cannot take it anymore. I’ve endured all my treatments so far. I endured the illness, the chemo […] I did everything like a little soldier. And I said, that’s it, no more. I’m taking back the power, and it’s not hormone therapy that will define who I am.”*

This shift in mindset was often accompanied by proactive behaviours aimed at improving physical and emotional well-being. Participants described engaging in complementary therapies, adopting self-care routines, and cultivating resilience. These actions were not only therapeutic but also symbolic of reclaiming identity and agency. For example, P15 used meditation to foster acceptance, while P10 and P24 described regaining control by shaving their heads before hair loss occurred or by purchasing accessories as a form of emotional expression.

Participants recognized that while medical treatments are the domain of professionals, they could still influence their recovery and quality of life through personal choices. This led to the adoption of integrative approaches combining conventional medicine with complementary practices. P27 spoke of “*gathering all her little weapons*,” while others developed personal philosophies centered on positivity and joy. Practices such as sport, writing, music, coaching, and meditation were frequently cited as tools for healing and self-empowerment.

P20 illustrated this proactive stance: *“Yes, physical pain, what helped me enormously was physiotherapy, clearly. […] When I left the hospital, I did not have a prescription. I had to ask for it myself. If I had not done physio, I do not know where I’d be today.”* She added, *“It’s important at some point to break free from that status of being a passive patient who just endures the consequences of what happens*.*”*

This form of self-empowerment was also framed as a message to others. P31 emphasized the importance of encouraging newly diagnosed women: “*To tell all these women who will face a diagnosis that they can participate and be co-authors of their care pathway […] and that these women can be in action*.” P29 echoed this sentiment: “*It’s a step toward healing, to be an actor, not a passive recipient. Even the treatments, saying: I’m going to do chemotherapy, I’m taking a step toward healing*.”

#### Patient-HCP relationship

The relationship between patients and healthcare professionals is a core theme of patient value, constituting the following subdimensions: encompassing communication practices (style and content), evolving relational dynamics, trust, shared decision-making, and therapeutic alliance.

A first subdimension of the patient-HCP relationship relates to both the style and content of communication practices as it can strongly impact patients’ reactions and state of mind: influence hope, fighting spirit, perceived fear, and the meaning of the treatment. Unfortunate words can deeply influence patients’ emotions, feelings and experiences. P18 described a traumatic moment when her surgeon bluntly stated, *“It’s massive and serious, but it’s huge,”* leaving her *“devastated, dissociated, unable to cry.”* The same participant also remembered the oncologist saying, “*I know some who made it through with this*.” She could not find hope in these words and described how it negatively impacted her fighting spirit: “*You want to ask yourself, “But what’s the point?”*

Clear, compassionate communication was often described as a turning point. P19 recalled receiving her diagnosis: *“Obviously, you cry all the tears in your body, but things have been said, have been put in a very clear, very human way.*”

Patients valued transparency, especially when facing uncertainty. P26 described a sense of relief upon receiving a clear diagnosis: *“So, that’s it, I have the words. A professional tells me so. Finally, I have my battle plan. It’s cool, I’m going to move forward.”*

Conversely, when HPCs avoided difficult words or used euphemisms, patients often felt confused or misinformed. P31, for instance, struggled to understand the implications of liver nodules: *“I’m not from the medical field—I did not know what that meant.”* Similarly, P2 questioned the rationale behind her treatment plan: *“I did not understand why we started with radiotherapy if I were told the organ would be completely removed.”*

These communication gaps sometimes led to patients receiving their diagnosis indirectly or only fully understanding their condition after repeated questioning. In contrast, open and honest discussions—even about poor prognoses—were seen as trust-building. P31 recounted how her oncologist explained the chronic nature of her metastatic cancer, the likelihood of resistance, and the sequence of treatment lines: *“She clearly explained everything. I left with trust in the medical team.”*

Providing complete and timely information also helped patients prepare for the physical and emotional consequences of treatment. When this was lacking, patients faced unexpected challenges. One participant accepted a new job contract just 1 month after surgery, unaware of the recovery limitations. Another was shocked by the extent of her surgical scarring, having received no prior explanation. P31 described the emotional impact of receiving permanent radiotherapy markers without consent: *“They tattooed me for life. It may seem minor to a doctor, but for a patient, it feels like being branded. At a time when you already feel like you have lost control of your body, it’s a lot.”*

These narratives underscore the profound influence of communication, not only on patients’ understanding and decision-making, but also on their emotional resilience and sense of dignity. The therapeutic relationship is not merely a clinical interaction; it is a deeply human connection shaped by empathy, clarity, and mutual respect.

A second subdimension relates to the dynamic and evolving character of the patient-HCP relationship. The initial connection between patients and HCPs often hinges on an intuitive “fit” between individuals, shaped by personality traits and subtle cues such as body language and first impressions. For example, one patient (P15) described the difficulty of choosing between two oncologists: *“I had chosen two oncologists. It was impossible to choose. I loved them both for very different reasons. […] There is one who is much rounder, much softer. The other one is much more go-getter, strict with me.”* This illustrates how patients perceive and respond to different relational styles, each offering distinct forms of support.

A third subdimension of the patient-HCP relationship relates to trust. P31 showed the immediate sense of trust that emerged during the first meeting: “*As soon as she opened the door, I do not know, there was something. It wasn’t necessarily in the words, body language matters. It was obvious that trust was there*.” Such moments underscore the importance of non-verbal communication and emotional resonance in establishing a foundation for therapeutic alliance.

Once trust is established, patients often feel empowered to relinquish control over the technical aspects of their care, allowing them to focus on areas where they retain agency. P19 reflected on this shift: *“I quickly felt confident after meeting the doctors. So, I immediately let go of the purely medical part. My role wasn’t there, it was elsewhere*.”

Trust also plays a critical role in how patients receive and process difficult information. P31 recounted a moment of clarity and reassurance during the explanation of a metastatic cancer diagnosis: *“She said it could be treated but not cured. She told me she would watch over me like milk on the stove and accompany me until her retirement. That gave me hope.*” This illustrates how compassionate communication can transform even distressing news into a source of comfort and motivation.

Conversely, the erosion of trust can severely disrupt the therapeutic relationship. P10 shared a pivotal moment: “*She decided to terminate my pregnancy, I could not continue with her […] she had not heard me.”* This breach of trust led to a change in provider, underscoring the importance of feeling heard and respected in clinical interactions.

The interplay between trust, information sharing, and empowerment is central to effective care. Patients consistently stressed the need for clear, personalized information to support informed decision-making. If shared decision-making was desirable for most podcast narrators, some raised the critical role HCP had to play to empower the patient without abandoning them to the burden of choice. P10 articulated this tension: “*I needed help. I was waiting for someone to guide me. But it never came. I was always told, ‘It’s up to you.’ But I had no evidence to decide*.”

This need for support is closely linked to goal-oriented care, which aligns treatment with patients’ personal context and life goals. Patients discussed a range of priorities, from maintaining a pregnancy to planning vacations or choosing surgical options. Yet asserting these preferences was often challenging.

A fourth subdimension of the patient-HCP relationship relates to the lack of shared-decision making. Participants still experienced hierarchical or paternalistic dynamics. P18 recounted a struggle to obtain a prophylactic mastectomy: “*I insisted. But he refused. At the end, he walked me to the door and said, ‘We’re a team. I’ll do it.’ I left feeling victorious. He did it like a favor*.” This narrative reflects both the persistence of gatekeeping and the emotional toll of advocating for one’s needs. It also reveals how patients often bear the burden of navigating care options, especially when their preferences diverge from normative expectations.

A fifth subdimension relates to the therapeutic alliance which was illustrated by self-reported patient experiences as a complex interplay of interpersonal connection, trust, communication, and systemic context. Patients’ voices illuminate the profound impact of being seen, heard, and supported—not only on their emotional well-being but also on their capacity to engage actively in their care. Participants’ narratives often revealed ambivalent feelings toward their oncologist, oscillating between deep attachment and moments of resistance. This emotional duality, sometimes described as a “love-hate” dynamic, reflects both recognition of the life-saving care received and the emotional toll of difficult interactions. One participant (P18) captured this tension poignantly: *“Every time I see him, I think—he saved my life. But at the same time, he said the harshest things I’ve ever heard.”* Despite these complexities, many participants spontaneously expressed appreciation for the care they received, often illuminating small gestures and attentions that made a significant difference in their daily lives.

These moments of human connection, often provided by nurses or support staff, were deeply valued. P2 described the emotional support she received: *“There were nurses who acted like a mother, like my daughter or my son would have. They were truly exceptional.”* Similarly, P19 underscored the importance of recognizing the medical team’s role in her recovery: *“What healed me were the doctors. […] Yes, there’s room for improvement in the relational aspect, but we must remember—there’s no second half if we do not make it through the first.”*

#### Quality of life

Quality of life emerged as a core theme of patient value capturing the multifaceted impact of cancer and its treatment on patients’ daily lives, encompassing both the physical and psychological dimensions of living with a chronic and life-threatening illness, the management of side effects, long-term consequences of treatment, and the use of integrative practices and self-care strategies. Rather than focusing solely on survival, participants articulated the importance of their lived experience, marked by discomfort, fatigue, and emotional strain, as central to their overall quality of life.

A first subdimension of quality of life relates to physical symptoms, including chronic pain, paraesthesia, fatigue, cognitive difficulties, etc. P22 vividly described the aftermath of treatment: “*After the treatments, you often hurt all over. You feel like you have been through a giant washing machine. You have aches in the morning, you creak like an old door*.” Other commonly cited side effects included mouth sores, weight loss, lymphedema, ageusia, sexual dysfunction, and surgical sequelae. P27 compared herself to “*a little granny*,” while P29 described the social withdrawal that followed her ostomy, stating she no longer dared to leave the house.

The persistence of pain and fatigue was particularly distressing. P17 shared, “*The pain is still there. It’s present. Lately, it’s been really difficult. The days are hard. […]”* P31 distinguished between fatigue and “fatigability,” explaining: “*Things I used to do without any problem now exhaust me—physically and cognitively. When there’s too much noise, too many people, it becomes overwhelming*.”

For some, the side effects were more unbearable than the disease itself, leading to frustration or even disgust toward treatment. P28 reflected, “*I accepted the illness, but I cannot accept the fact that I’m becoming physically diminished*.” P2 described her body reaching a breaking point: “*The body says stop! The veins burst. They tried pills. As soon as they went in, they came out. I could not tolerate anything anymore*.” P28 added, “*I felt disgusted by the treatment. I stood in front of the mirror with my IV bag and imagined the product entering my body. I trembled with disgust*.”

A second subdimension relates to the psychological toll of survivorship and the fear of recurrence. P22 recalled, “*I woke up thinking about cancer. I went to bed thinking about cancer. I thought about it all day, every day. And people did not understand, they expected me to be better*.” Changes in self-image and social life compounded these experiences. P17, for example, avoided physical contact and intimacy due to her surgical scars: “*It’s complicated. I refused. I did not want anyone to touch me. I did not want anyone to see that scar*.”

Participants noted the need to balance treatment efficacy with tolerable side effects to maintain adherence and preserve dignity. P31 explained, “*There are markers checked at every blood test. It’s a compromise between treatment effectiveness, which is currently very good for me, and my quality of life. We have to find that balance*.” P32 echoed this, stating: “*The patient’s quality of life must be factored into the benefit–risk equation. I’m living proof that, for example, hormone therapy can completely undermine treatment adherence*.”

A third subdimension relates to integrative practices and self-care strategies as a way to navigate physical symptoms and psychological toll. These included physical activities (e.g., sport, diving, yoga, and boxing), mind–body techniques (e.g., meditation, mindfulness, acupuncture) and holistic approaches (Ayurveda, dietary changes). These practices were not only therapeutic but also fostered emotional resilience and a renewed connection with the body. P15 described sport as her “*daily mantra, my antidepressant, my anti-fatigue*.” P40 spoke of “*pleasure through all the senses, a body–mind tandem*.”

The therapeutic relationship with complementary care providers was often described as more emotionally supportive than traditional medical encounters. P17 shared, “*I work with a reflexologist who has almost become a friend. She was there for my body, my suffering, and also psychologically*.” P18 found reassurance in alternative spaces: “*Doing other things, seeing other people who reassured me, because the medical environment itself was not reassuring. I saw helplessness in my doctors’ eyes, and that was really hard*.” P21 reflected on the healing power of diving: “*It helped me let go. It made me dream. It really did me good.*”

Ultimately, these approaches contributed not only to symptom management but also to a broader process of self-reconciliation and healing. P22 spoke of learning to forgive her body, while P31 described the importance of “*being gentle, listening to oneself, and stopping the guilt*.” These narratives underscore the importance of addressing quality of life as a core component of cancer care that extends beyond survival to encompass dignity, agency, and emotional well-being.

#### Finding opportunity in cancer

Finding opportunity in cancer emerged as a core theme out of patients’ self-reported experiences, leading to personal transformation, priorities in life, and life choices. For many participants, cancer marked a profound turning point, an experience that, while undeniably painful, also prompted deep personal transformation.

A first sub-dimension of finding opportunity in cancer relates to personal transformation. Although cancer was described as a traumatic and life-altering event, participants frequently framed it as an opportunity for growth, learning, and redefinition of self. A recurring narrative was the discovery of greater self-awareness and self-compassion. Participants described learning to care for themselves with more kindness, reconnecting with simple pleasures, embracing acceptance, and adopting a “carpe diem” mindset. P28 reflected, “*I took off the blinders. I have much more love and empathy. I’m much more at peace with myself. All those silly questions that had no value in life have disappeared*.” Similarly, P18 shared, “*I used to be preoccupied. The illness gave me the present moment*.”

A second subdimension of finding opportunity in cancer relates to setting priorities in life. Cancer was seen as a catalyst for reorienting one’s life. The experience prompted many to reassess their values and make deliberate choices about how they wanted to live. P19 described this as a “*secondary benefit*” of the illness: “*It reshuffles the deck in your personal life, professional life, and family life. We now express affection in ways that are life-changing*.” For some, this shift led to major life changes: returning to school, changing careers, healing past wounds, or pursuing long-held dreams. As P18 put it, “*It was a dream, and I allowed myself to pursue it*.”

A third subdimension of finding opportunity in cancer relates to life choices. Patients described the illness as a powerful justification to reclaim lost passions or explore new paths. P20 explained, “*I started doing things I never had time for before. […] The illness gave me a huge excuse to reconnect with things I used to love but had lost sight of.”*

For those who changed their professional trajectory, this often reflected a search for meaning, purpose or a desire to help others. Several participants reported discovering tools during their own journey and feeling compelled to share them by becoming coaches, patient partners, or founding associations and OHCs to raise awareness and foster connection.

P20 expressed this transformation clearly: “*I want to do something with this illness and let it be reflected in my professional practice.*” These actions were not only empowering for the individuals themselves but also reflected a broader commitment to fostering autonomy and resilience in others. Ultimately, these narratives underscore that cancer affects the whole person, not only physically, but emotionally, socially, and existentially. While the clinical focus is often on survival and treatment outcomes, participants’ experiences highlight the potential for post-traumatic growth and the redefinition of life’s meaning in the aftermath of illness.

For many, cancer was a transformative experience, fostering self-awareness, renewed purpose, and personal growth. Participants often redefined priorities and found opportunities to help others, accentuating post-traumatic growth beyond survival.

#### Respecting individuality in the cancer experience

Respecting individuality in the cancer experience is a core theme of patient value comprised of subdimensions as patients’ personality, the importance of timing and their personal environment.

As a first subdimension, personality emerged as an important factor in the cancer experience. While some described feeling overwhelmed by the sudden onset of illness, *“swept into a whirlwind from one day to the next,”* others found comfort in the immediacy of action. For example, P21 shared, “*From a psychological standpoint, I did not experience long moments of uncertainty or diagnosis. I was immediately swept into treatment*,” expressing relief at being spared the anxiety of waiting. This diversity of experience underscores the importance of respecting each patient’s unique trajectory—before, during, and after cancer. As P21 reflected, “*That’s what I wanted to bring into this idea of a travel guide: to draw your own path during and after the journey*.” Participants stressed that there is no single way to experience illness, and that care should be adapted to the individual’s rhythm, environment, and emotional readiness.

A second subdimension relates to timing. Participants described how their emotional and informational needs evolved over time, and how their perception of time itself shifted depending on the phase of treatment. P21 illustrated this vividly: “*There are moments that feel very long. And at the same time, after a chemo, there are blank periods, the mind is foggy, you do not know what day it is, whether it’s early or late, you feel a bit disconnected.*”

A third subdimension relates to patients’ personal environment as an extension of individuality including family dynamics, professional responsibilities, and social networks, significantly shaping how participants experienced and processed their illness. Individual circumstances, such as being pregnant, the professional situation, or knowing someone in the medical field, could all influence the cancer experience. P18 recounted how a friend fast-tracked her into treatment: “*I texted my friend who’s a gynecologist, and she said, ‘I’m not reassured, I’m getting you an appointment for tomorrow at 9 a.m.*”

Participants expressed how contextual factors, such as emotional support, living conditions, and mental preparedness, were critical in shaping their experience. P21 noted, “*All the stages we talk about are lived differently by each person. […] There will never be identical outcomes. Psychological factors, the role we play as fighters, how we are supported, where we live, and how we feel are all determining elements. And in a way, that’s reassuring: even with the most severe diagnosis, there is always hope*.”

P29 added, “*We all arrive with our own baggage. We all face illness differently. I’m used to managing large-scale projects, so I approached the illness like a project. My friends joked that I delivered my treatment like I deliver my projects—on time. So yes, mindset matters*.”

This individuality also has implications for the therapeutic relationship, particularly regarding communication. Participants mentioned that HCPs should tailor their communication to the patient’s stage in the care pathway, considering emotional and cognitive availability. Needs fluctuated over time, and key discussions may need to be revisited to ensure understanding and alignment. Participants underscored the importance of preparing patients and creating space for evolving dialogue. The timing and setting of communication were also seen as critical. P18 recounted a distressing moment: “*I was in the operating room, and my surgeon stuck his head in and said, ‘By the way, we have the results of the sentinel lymph node. Frankly, it does not look great, so I’ll take everything out.’ He told me that right then. And I’m like, ‘Ah, okay’ […] I fell asleep with that*.”

Preferences for information delivery varied widely. Some patients wanted to be fully involved in shared decision-making, while others preferred a gradual approach to avoid emotional overload. P2 explained, *“So no, I had not been told about an ostomy for life, certainly not, otherwise I do not think I would even have gone through with the surgery. All this was announced as we went along, of course, so as not to scare me.”*

## Discussion

This qualitative study examines cancer patients’ self-reported experiences, shared spontaneously on a podcast platform within an OHC to identify what patients themselves define as valuable within their cancer journey. While patient experience has gained increasing attention in oncology research, spontaneous patient narratives remain underexplored, particularly those shared outside formal research settings.

Interestingly, despite the fundamentally different contexts and intents, the core themes that emerged from these self-reported podcast narratives closely mirror those identified in researcher-led studies (Horicks and Coppieters, 2021; Vaz-Luis et al., 2022; Taylor, 2024). In traditional research settings, patients respond to (semi-) structured interviews or surveys designed to elicit specific information. In contrast, the participants in this study shared their stories spontaneously, primarily to connect with peers or to reach a broader audience. This distinction underscores the authenticity and emotional richness of the data, offering a complementary perspective to more conventional methodologies.

**Figure 1:**
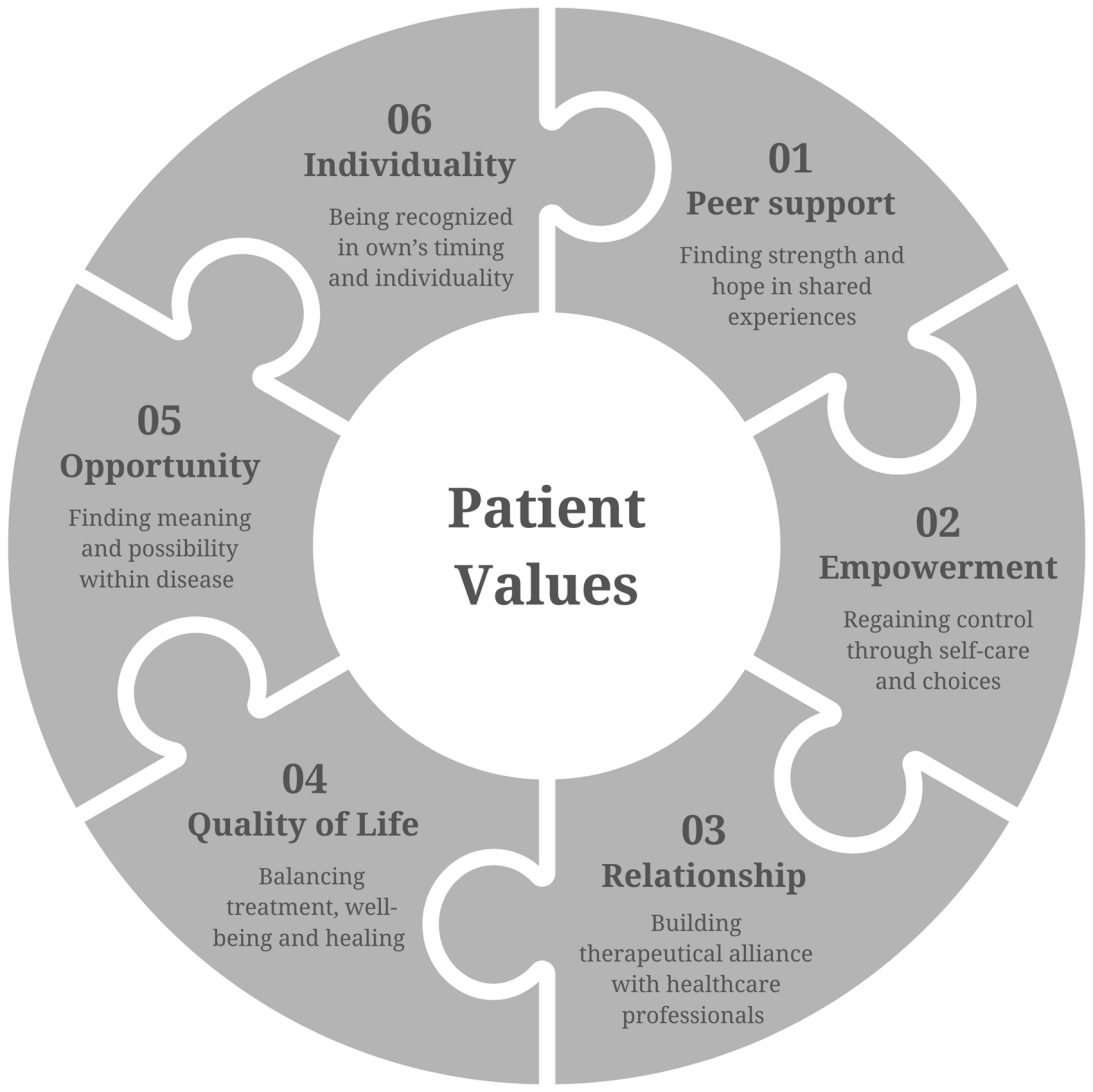
General overview of patient values identified in self-reported patient narratives.

We focused specifically on the concept of patient value in oncology. The podcast narratives were analyzed through this lens, revealing six intertwined core themes of patient value that patients communicate and reinforce within their peer community (Figure 1): (i) peer support, (ii) empowerment, (iii) relationship with HCPs, (iv) quality of life, (v) cancer as opportunity, and (vi) individuality. The findings of this study highlight the deeply personal, complex, and multifaceted nature of the cancer experience. Patients navigate illness by drawing strength from values that are meaningful and specific to them.

### The value of support, especially among peers

A recurring value across the narratives is the importance of emotional support, particularly from one’s social circle. However, as identified in previous studies (Northouse et al., 2012; Wang and Li, 2024), patients often reported challenges in communicating with family members, driven by a desire to protect loved ones or guilt. In this context, peer support emerged as a vital resource. Connecting with others who had undergone similar experiences provided emotional validation, practical advice, and a sense of solidarity. It helped normalize suffering, reduce isolation and foster hope (Skirbekk et al., 2018). Several participants described how helping others gave new meaning to their own experience with cancer. This reciprocity, being both supported and supportive, was seen as a powerful coping mechanism. Ziegler et al. (2022) found that peer support significantly increased psychological empowerment and was associated with broader patient-reported benefits (Ziegler et al., 2022). Similarly, Skirbekk et al. (2018) brought the value for peer supporters to the foreground, noting that the act of helping others enhanced their own resilience and sense of purpose.

However, as far as OHCs themselves are concerned, patients are also cautioned against the potential downsides. Exposure to distressing stories, misinformation or overwhelming emotional content could be detrimental, underscoring the need for guidance in navigating digital communities. While face-to-face and online peer support generally receive high satisfaction ratings (Huber et al., 2018), evidence about psychological impact is mixed (Jablotschkin et al., 2022; Kiemen et al., 2023). For instance, Høybye et al. (2010) reported that participation in an internet-based peer support group following rehabilitation did not significantly improve mood disturbance. However, it may have strengthened patients’ fighting spirit through social interaction.

### The value of (regaining) control

A prominent theme emerging from the podcast narratives is the value patients place on regaining control over their illness and their lives through empowerment. Many participants described the experience of cancer as one of passivity and being acted upon rather than acting. Patients sought to move from enduring their care pathway to actively shaping it.

This reappropriation of control frequently occurred through therapeutic alliance, shared decision-making, and self-care practices. These mechanisms enabled patients to reassert their autonomy and participate meaningfully in their care. The concept of patient empowerment, long recognized in the literature, has been associated with increased self-efficacy and improved health outcomes, including better psychological well-being (Fumagalli et al., 2015; Kershaw et al., 2015).

However, true empowerment demands appropriate support and knowledge. Several participants described the difficulty of being asked to make decisions without sufficient information or guidance. This highlights the importance of tailored communication and the need for HCPs to adapt their approach to each patient’s readiness and preferences. These findings echo calls in the literature for enhanced training in shared decision-making and patient-centered communication (Fallowfield et al., 2025).

### The dual nature of the patient-HCP relationship

The relationship with HCPs emerged as both a source of strength and a site of tension. Notably, complementary care providers were often perceived as offering more emotionally present, time-rich, and human-centered care. This contrast invites reflection on the relational dimensions of healing, suggesting that trust, empathy, and continuity are essential components of holistic, patient-centered and value-based care (Prip et al., 2018; Sanders et al., 2021; Elkefi and Asan, 2023).

Importantly, communication breakdowns were not always attributed to negligence. Participants acknowledged the systemic constraints clinicians face, including time pressure and administrative burdens (Zota et al., 2023). Nonetheless, these limitations can create information gaps that compromise trust and hinder patient engagement. Bridging the gap between clinical intention and patient experience is essential to align medical recommendations with the lived realities of care (Vermeir et al., 2015).

### The lasting impact of cancer on quality of life

Beyond the clinical trajectory, participants emphasized the profound and enduring effects of cancer on their quality of life. Physical sequelae, such as fatigue, pain or changes in appearance, often persisted long after treatment ended, affecting self-image, socio-professional reintegration, and autonomy. These experiences underscore the importance of viewing survivorship not merely as a return to health, but as a redefinition of life with and after cancer.

From the patient’s perspective, healthy life years extend beyond survival and confer value through the quality, autonomy, and meaning embedded in everyday living (Wang et al., 2025). Participants expressed a strong desire to live a life that is not only longer but also pleasant, purposeful, and aligned with their values. This reinforces the need for care models that prioritize patient-defined outcomes.

### Finding opportunity in cancer

Beyond the evident struggle, many participants described cancer as a transformative opportunity, one that prompted a reorientation of life values, priorities, and ways of being. For some, this experience was akin to a “second chance,” fostering deeper self-awareness, gratitude, and a more intentional approach to living. This re-evaluation aligns with the intrinsic human drive for growth and learning, becoming a powerful catalyst for self-reflection and adaptation in the aftermath of illness. This adaptive drive enabled patients to navigate uncertainty and engage actively in their recovery, echoing findings from studies on breast cancer patients (Rager, 2003). Similarly, Bilodeau et al. (2022) showed that heightened awareness of time’s passage encouraged patients to proactively confront post-cancer challenges by recognizing emotional triggers, engaging in reflection, and embracing trial and error as a learning process (Bilodeau et al., 2022). These narratives illustrate how illness can be transformed into a source of meaning, agency, and empowerment, both personally and collectively.

### The individuality of the cancer journey and the meaning of time

A central insight from this study is the irreducible individuality of the cancer experience. Participants indicated that no single care or communication strategy model can be universally applied. Each person approaches illness through the lens of their unique life history, values, personality, and emotional resources, as previously noted (Mohlin and Bernhardsson, 2021). Temporal experiences also varied widely, influenced by treatment phases, care pathways and social contexts.

These findings underscore the value of personalized care pathways that respect not only clinical profiles but also psychosocial contexts and individual rhythms of adaptation. In this context, time emerges as both a symbolic and practical construct, shaping decision-making and emotional processing throughout the cancer journey (Thorne et al., 2009; Wieringa et al., 2024). There is no one-size-fits-all approach; each patient interprets their situation differently and prioritizes life goals in their own way.

This complexity intersects with communication practices, in which subtle nuances can significantly affect patient experiences. It also reflects the interplay between patients’ personal timelines, unconscious biases, and the personalities of both patients and HCPs. The ability to perceive and respond to individual communicative needs is deeply embedded in cultural norms and requires targeted training to enhance both the relevance and effectiveness of care (Moore et al., 2018). Strengthening communication skills among HCPs and fostering patient engagement are essential for promoting health literacy, empowerment, and shared decision-making (Merckaert et al., 2015).

### Strengths and limitations

A key strength of this study lies in its focus on cancer patients’ self-reported experiences, shared independently of HCPs or researchers. This approach offers valuable insights for future research aiming to integrate patient perspectives, derived directly from narratives, alongside researcher-led assessments and clinical data. The transdisciplinary collaboration, which includes researchers from diverse fields and the patient-founder of the peer community, further enhances the relevance and depth of our findings.

However, limitations must be acknowledged. The selected podcast sample may be subject to bias for three main reasons. First, the involvement of the patient-founder in the preselection process may have influenced the thematic focus of the narratives. Participants may have been selected to align with the podcast’s intended audience and the patient-founder’s goal of sharing positive, useful, and inspiring experiences.

Secondly, the study sample is limited by its gender distribution and cultural context, comprising solely French-speaking participants based in Europe. This may limit the transferability of insights to other cultural or linguistic contexts. Patients’ values, experiences, and communication preferences may differ across regions, healthcare systems, or languages, and these differences should be considered when generalizing these results.

Third, the use of qualitative material not specifically generated for research entails inherent constraints related to representativeness and control over context. Participation in a publicly available podcast requires openness, communicative confidence, and a willingness to share personal experiences that may not be characteristic of the broader population of cancer patients. This visibility likely introduces a degree of self-selection bias, favoring participants who are comfortable articulating their illness journey and may present more reflective, expressive, or outward-focused perspectives. As a result, the dataset likely shows themes of positivity, learning, and resilience.

While this introduces a degree of subjectivity, it remains consistent with the study’s primary objective: to explore self-reported patient experiences within a peer-to-peer framework and to understand what patients value and wish to transmit to others. In this context, the subjective nature of the data is not a limitation but rather a reflection of the study’s core purpose.

Here, we chose to focus our analysis on patients’ episodes because the central aim of the paper was to explore the values that patients share with, and for patients within a peer-support community. A follow-up study could extend this work by analyzing the narratives of HCPs and conducting a “mirror analysis” that compares and contrasts both perspectives. Such an approach would provide a more comprehensive understanding of how patient and professional voices align or diverge in articulating what is most valued in the cancer journey.

### A patient’s perspective on the study

From the patient-founder’s perspective, this study reveals the importance of structuring subjective experiences to inform care practices meaningfully. She emphasized that moving from the passive position of being a patient to engaging in an in-depth analysis of “patient value” demonstrated how highly personal information can be included in scientific material with the potential to improve care pathways. The values identified through this process resonate with those consistently emerging in her podcast and interactions with patients from her OHC: feelings of isolation, the search for meaning, the need for emotional validation, and the contagious power of hope. She recognized that the spontaneity and authenticity of narratives could be preserved while still being studied rigorously. Precisely because spontaneous narratives resist being prematurely confined to predefined categories, they offer unique and valuable insights into what truly matters to patients.

### Reframing patient value

This study highlights that patient value extends far beyond biomedical outcomes or financial metrics. For many participants, value was rooted in reclaiming autonomy, redefining life priorities, and engaging in meaningful action—whether through personal growth, peer support, or professional reinvention. Empowerment emerged as both a process and an outcome, shaped by individual trajectories, contextual factors, and emotional resilience. Illness often prompted a re-evaluation of self, relationships, and time, revealing deeply personal understandings of what it means to live well. Our study explored what patients spontaneously expressed as most valuable at a micro and individual level. However, the lack of conceptual clarity in the literature on “patient value” impedes understanding of what constitutes value-based care from the patient’s perspective.

Understanding self-care practices is essential, especially for individuals with chronic conditions like cancer, who make ongoing decisions that directly affect their well-being, autonomy, and health-related quality of life. This is increasingly captured through patient-reported outcomes (PROs) (Deshpande et al., 2011). Clarifying the concept of patient value can pragmatically inform cancer care and research, guiding the development of more effective care plans, assessments and interventions that support patient autonomy and align care with what truly matters to individuals (Karimi-Dehkordi et al., 2019).

These findings call for a broader, more holistic interpretation of patient value, one that integrates clinical goals with the social, psychological, and existential dimensions of health.

## Conclusion

Drawing on cancer patients’ self-reported experiences within an OHC podcast, this study highlights the shared desire for a holistic approach to care that honors the full spectrum of human experience. Patients highlighted the added value of peer-support communities in fostering therapeutic relationships, validating individual timelines, and creating environments grounded in empowerment, mutual support, and solidarity. These exchanges reflect a truly patient-centered model that encompasses not only the psychological and relational dimensions of illness but also its existential significance.

Future research and policy should continue to prioritize patients’ voices and integrate their most valued considerations throughout their cancer pathway. Doing so will help ensure that care systems are equipped to address the complex, multidimensional challenges of cancer care.

OHCs, used daily by millions worldwide, represent a promising yet underexplored resource. Additionally, the impact of patient testimonials on individuals whose experiences diverge from the dominant narratives within these communities warrants further investigation.

The community studied in this research is strongly oriented around themes of hope, mutual support, and resilience. Yet, it remains uncertain how individuals who do not identify with these values might perceive or respond to such narratives. Further research is essential to assess the actual impact of these communities on users’ mindsets, emotional well-being, and health outcomes, both for active members and those who engage more passively.

## Data Availability

Publicly available datasets were analyzed in this study. This data can be found here: https://cancer-je-gere.blog/podcast/.
