# Supplementary material for: Self-reported patient experiences in a peer-support community: What do cancer patients value?
Source: Front Psychol. 2026 Mar 5;17:1724101. doi: 10.3389/fpsyg.2026.1724101 (PMC13003601; doi:10.3389/fpsyg.2026.1724101)
Supplement: Supplementary file 1 [file Supplementary_file_1.docx]

**Supplementary file 1**. Inclusion and exclusion criteria

For the manuscript Horicks et al. *“Self-Reported Patient Experiences in a peer-support community: what do cancer patients value?”*

| Inclusion criteria |
| --- |
| Podcast in ‘Naître princesse, devenir guerrière’ |
| Cancer patient voice  Adult narrator |
| Self-reported narrative |
| Exclusion criteria |
| Professional voice  Entrepreneurial focus  Singular subject |
