# Supplementary material for: Self-reported patient experiences in a peer-support community: What do cancer patients value?
Source: Front Psychol. 2026 Mar 5;17:1724101. doi: 10.3389/fpsyg.2026.1724101 (PMC13003601; doi:10.3389/fpsyg.2026.1724101)
Supplement: Supplementary file 2 [file Supplementary_file_2.docx]

**Supplementary file 2. Codebook**

For the manuscript Horicks et al. *“Self-Reported Patient Experiences in a peer-support community: what do cancer patients value?”*

| **Code** | **Subcode** | |
| --- | --- | --- |
| 1. Patient background |  |  |
| 2. Healthcare System | 2.1 | Lack of resources |
|  | 2.2 | Healthcare system Culture |
|  | 2.3 | Paternalism |
| 3. Concepts of care | 3.1 | Patient centricity |
|  | 3.2 | Holistic approach |
|  | 3.3 | Personalized treatment |
|  | 3.4 | Patient advocacy |
|  | 3.5 | Patient involvement |
|  | 3.6 | Patient partnership |
|  | 3.7 | Shared decision making |
|  | 3.8 | Informed decision |
|  | 3.9 | Patient empowerment |
|  | 3.10 | Integrative care |
|  | 3.11 | Goal oriented care |
| 4. Pathway description | 4.1 | Prevention |
|  | 4.2 | Pathology description |
|  | 4.3 | Diagnosis description |
|  | 4.4 | Pre-diagnosis description |
|  | 4.5 | Screening (in diagnosis) |
|  | 4.6 | Treatment description |
|  | 4.7 | Changing HCP |
|  | 4.8 | Second Opinion |
|  | 4.9 | CAM's |
|  | 4.10 | Side-effects |
|  | 4.11 | Urge |
|  | 4.12 | Collaboration-Fragmentation |
|  | 4.13 | relapse |
| 5. Communication | 5.1 | Giving info to the patient |
|  | 5.2 | Bad news announcement |
|  | 5.3 | HCP communication training |
|  | 5.4 | HCP communication skills |
|  | 5.5 | Communication problems |
|  | 5.6 | Importance of the timing of communication |
|  | 5.7 | Transparency |
|  | 5.8 | Mutual understanding |
| 6. Relationship HCP-P | 6.1 | Disbalanced relationship |
|  | 6.2 | Respect the patient choice |
|  | 6.3 | Therapeutic adherence |
|  | 6.4 | HCP projection |
|  | 6.5 | Support-help from HCP |
| 7. Micro Level Patients | 7.1 | Patient Perspective |
|  | 7.2 | Refusal of treatment |
|  | 7.3 | Medical Burn-out |
| 8. Subjective elements about the patient | 8.1 | Resilience |
|  | 8.2 | Positivity-negativity |
|  | 8.3 | Give meaning to the disease |
|  | 8.4 | Dreams |
|  | 8.5 | Survival mode |
|  | 8.6 | (self)Trust (loss of) |
|  | 8.7 | Reassurance |
|  | 8.8 | Cancer as an opportunity |
|  | 8.9 | (Loosing) Hope |
|  | 8.10 | Being usefull |
|  | 8.11 | Dealing with new roles |
|  | 8.12 | Enjoying small things in life |
|  | 8.13 | Injunction |
|  | 8.14 | Future perception |
|  | 8.15 | Individuality |
|  | 8.16 | Importance of appearance |
|  | 8.17 | Patient beliefs |
|  | 8.18 | Patient preferences |
|  | 8.19 | Patient instinct |
|  | 8.20 | Spirituality |
|  | 8.21 | Anticipation |
|  | 8.22 | Self-esteem |
|  | 8.23 | Comparison to others |
|  | 8.24 | Self-image |
|  | 8.25 | Prejudice |
|  | 8.26 | Feminism |
|  | 8.27 | Panick |
|  | 8.28 | Emotional shock-tsunami |
|  | 8.29 | Denial |
|  | 8.30 | Loneliness |
|  | 8.31 | Feeling (un)heard-(not)understood |
|  | 8.32 | Guilt |
|  | 8.33 | Empathy |
|  | 8.34 | Emotional rollercoaster |
|  | 8.35 | Patients fears |
|  | 8.36 | Gratitude |
|  | 8.37 | Uncertainty |
|  | 8.38 | trigger |
|  | 8.39 | depression-victimization |
|  | 8.40 | (Non) helping factors |
|  | 8.41 | Letting go - control |
|  | 8.42 | (ab)normality feeling |
|  | 8.43 | (emotionnal) distance |
| 9. Objective elements about the patient & their environment | 9.1 | Pregnancy/motherhood |
|  | 9.2 | Patient indiv. Circumstances |
|  | 9.3 | QoL (physical aspects) |
|  | 9.4 | Challenges/side effects |
|  | 9.5 | Body-mind link |
|  | 9.6 | Patient needs (fluctuation of) |
|  | 9.7 | Shift in life |
|  | 9.8 | Coping-mechanisms |
|  | 9.9 | Priority settings (personal for the patient) |
|  | 9.10 | Professionnal situation |
|  | 9.11 | Financial problems |
|  | 9.12 | Life adaptations |
|  | 9.13 | Self-care |
|  | 9.14 | Timing-evolution with time |
|  | 9.15 | Reappropriate new self |
|  | 9.16 | Survivorship |
| 10. Entourage patient | 10.1 | Importance of family |
|  | 10.2 | (De)burden family |
|  | 10.3 | Support of family |
|  | 10.4 | Role of partner |
|  | 10.5 | Role of children |
| 11. Support | 11.1 | Support of peers |
|  | 11.2 | Support through social media |
|  | 11.3 | Helping others |
|  | 11.4 | Support-comfort entourage |
